# Uncovering the hydride ion diffusion pathway in barium hydride via neutron spectroscopy

**DOI:** 10.1038/s41598-022-10199-8

**Published:** 2022-04-13

**Authors:** Eric Novak, Luke Daemen, Anibal Javier Ramirez-Cuesta, Yongqiang Cheng, Robert Smith, Takeshi Egami, Niina Jalarvo

**Affiliations:** 1 Department of Materials Science and Engineering, University of Tennessee, Knoxville, TN 37996 USA; 2 Neutron Sciences Directorate, Oak Ridge National Laboratory, Oak Ridge, TN 37831 USA; 3 Jülich Centre for Neutron Science, Forschungszentrum Jülich GmbH, 52425 Jülich, Germany; 4 Computing & Computational Sciences Directorate, Oak Ridge National Laboratory, Oak Ridge, TN 37831 USA; 5 Materials Science and Technology Division, Oak Ridge National Laboratory, Oak Ridge, TN 37831 USA; 6 Department of Physics and Astronomy, University of Tennessee, Knoxville, TN 37996 USA

**Keywords:** Materials for energy and catalysis, Energy

## Abstract

Solid state materials possessing the ability for fast ionic diffusion of hydrogen have immense appeal for a wide range of energy-related applications. Ionic hydrogen transport research is dominated by proton conductors, but recently a few examples of hydride ion conductors have been observed as well. Barium hydride, BaH_2_, undergoes a structural phase transition around 775 K that leads to an order of magnitude increase in the ionic conductivity. This material provides a prototypical system to understand hydride ion diffusion and how the altered structure produced by the phase transition can have an enormous impact on the diffusion. We employ quasielastic and inelastic neutron scattering to probe the atomic scale diffusion mechanism and vibrational dynamics of hydride ions in both the low- and high-temperature phases. Jump lengths, residence times, diffusion coefficients, and activation energies are extracted and compared to the crystal structure to uncover the diffusion pathways. We find that the hydrogen jump distances, residence times, and energy barriers become reduced following the phase transition, allowing for the efficient conduction of hydride ions through a series of hydrogen jumps of length *L* = 3.1 Å.

## Introduction

Hydrogen is a key alternative energy source in the effort to reduce the dependence on fossil fuels. Metal hydrides have the ability for the fast and reversible uptake and release of large quantities of hydrogen, making them candidates for energy storage devices such as fuel cells and batteries. While most metal hydride studies have focused on the lighter weight hydrides for hydrogen storage applications, the heavy alkaline earth hydrides of barium, calcium, and strontium have received much less attention. Recently, it was found that this group exhibits a rare form of ionic conduction of hydride ions^1,2^. Along with these few metal hydrides, pure hydride ion diffusion (in the absence of electronic conduction) has only been reported for oxyhydrides^3–5^. In this work, we study barium hydride, BaH_2_, which exhibits fast ionic conduction of hydride ions due to a structural phase transition at high temperatures. In fact, the conduction mechanism is so efficient that the ionic conductivities are higher than most of the leading proton and oxide ion conductors^1^. The diffusion of hydrogen through the crystal structure is a key rate-limiting step for use in energy devices. Hence, fast ionic transport is a highly desirable property to produce efficient devices. Therefore, understanding the atomic scale diffusion mechanism in this interesting system can improve our understanding of solid-state hydrogen transport and help advance the development of metal hydrides with improved kinetics.

Numerous studies have been devoted to the investigation of the crystal structure of barium hydride^1,6–9^. The crystal structure, commonly known as cotunnite, has orthorhombic symmetry with the space group Pnma (62). The lattice parameters for BaD_2_ have been determined by neutron powder diffraction to be *a* = 6.7824(1) Å, *b* = 4.1605(1) Å, and *c* = 7.8432(1) Å at *T* = 298 K^1^. The hydrogen in this system resides on two crystallographically distinct 4c sites, referred to as H(1) and H(2). Barium hydride has been reported to have significant hydrogen sub-stoichiometries, particularly BaD_x_ with *x* ~ 1.8, where the large concentration of vacancies leads to a vacancy-mediated transport mechanism^1,2,10^. A structural phase transition occurs around 773 K from the orthorhombic phase to a high symmetry hexagonal (Ni_2_In-type) phase with the space group P6_3_/mmc (194)^1,6,9^. The hexagonal unit cell has lattice parameters of *a* = 4.4571(2) Å and *c* = 6.7230(4) Å at *T* = 883 K^1^. Neutron powder diffraction measurements have suggested that the H(1) site is split in the hexagonal phase, where it deviates from the high-symmetry 2d site and instead resides on the 4f site^1,9^. Upon the phase transition, the ionic conductivity increases by more than an order of magnitude from 5^.^10^–3^ S/cm at 693 K to 1^.^10^–1^ S/cm at 823 K^1^, which is at the higher end for ionic conduction values in solids at similar temperatures. Among the best known ionic conductors, acceptor doped solid oxides, such as BaZrO_3_^11^, BaPrO_3_^12^, and BaCeO_3_^13^, have conductivities ranging from 10^–2^ S/cm to 10^–1^ S/cm in this temperature range. The hexagonal phase of BaH_2_ can also be formed at room temperature at high pressures around 2.5 GPa^14–19^. Zhang et al. recently reported that this high pressure phase also exhibits ionic conduction around 10^–4^ S/cm^17^.

In this work, we investigate both the low temperature orthorhombic and the high temperature hexagonal phases of BaH_2_ using neutron spectroscopy, which is a powerful tool to study hydrogenous materials. Hydrogen has an exceptionally large incoherent neutron scattering cross section; e.g. in BaH_2_, 99.9% of the incoherent signal arises exclusively from hydrogen. Quasielastic incoherent neutron scattering (QENS) is used to probe the ionic transport of the hydride ions on the atomic scale, while powder inelastic neutron scattering (INS) is applied to explore the temperature dependence of the vibrational modes of the hydrogen atoms.

## Methods

A thorough account of the experimental details and data analysis methods is included in the supporting information (SI), but a summary is presented here. Polycrystalline BaH_2_ was purchased with a reported purity of 99.7% and measured without further modification. For the neutron powder diffraction measurement, BaD_2_ was synthesized through a direct reaction of barium metal and deuterium gas. 6 g of Ba was sealed in an autoclave with a graphite gasket. This was reacted at *T* = 180 °C and a pressure of 27 bar D_2_ gas for 24 h. The product was light grey in color and reactive to water. The phase purity of the samples was confirmed by X-ray powder diffraction.

QENS measurements were performed using the neutron backscattering spectrometers BASIS at Oak Ridge National Laboratory (ORNL)^20^ and HFBS at the National Institute of Standards and Technology (NIST)^21^. These spectrometers can probe diffusive motions of hydride ions on time scales ranging from ps to ns, and on an Å length scale. The BASIS experiment used the Si (111) crystal analyzers for elastic scan measurements and Si (311) crystal analyzers for the QENS measurements. Si (111) and Si (311) analyzers provide an elastic energy resolution of 3.5 and 15 μeV (full-width at half maxima (FWHM)), respectively. The elastic energy resolution of HFBS is 0.8 μeV, which provides access to probe slower motions than accessible at BASIS. The QENS data fits were performed using the *QCLIMAX* package within *ICE-MAN*, the Integrated Computational Environment-Modeling & Analysis for Neutrons^22^. Inelastic neutron scattering measurements were performed at VISION at ORNL^23^ and data reduction was carried out using *Mantid*^24^. Neutron powder diffraction was measured at NOMAD at ORNL^25^.

## Results and discussion

**Figure 1:**
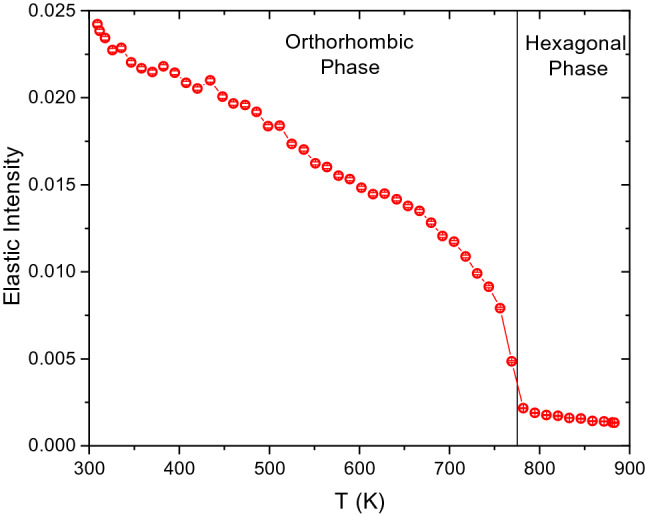
Elastic intensity scan measured at BASIS. The data is averaged over a *Q*-range of 0.5 – 1.5 Å^-1^.

Although the quasielastic neutron scattering spectra of BaH_2_ is dominated by the incoherent scattering of hydrogen, structural Bragg peaks arising from the coherent signal are present in the studied *Q*-range as well. Here we will only discuss the incoherent part of the spectra and omit the analysis of the Bragg peaks, which mainly influence the elastic intensity. The QENS spectra contains two major parts; the elastic peak and the quasielastic broadening of the elastic peak. With respect to the instrumental resolution, the immobile species contribute to the elastic peak, and the quasielastic broadening represents the diffusing species. The incoherent dynamic scattering function can be given as^26^,

1 $${S}_{inc}\left(Q,E\right)=f\left[X(Q)\delta \left(E\right)+(1-X(Q))\frac{\Gamma (Q)}{{\omega }^{2 }+{\Gamma }^{2}(Q)}\right]\otimes R\left(Q,E\right)+B\left(Q,E\right)$$

where the elastic contribution is represented with a *δ* function, numerically convoluted with the experimentally determined resolution function, *R(Q,E).* The resolution function is typically measured at a low temperature where all the diffusive motions on the measured time scale are frozen. The quasielastic broadening can be modelled using Lorentzian functions. The half-width at half-maxima (HWHM), *Γ*, of each Lorentzian is inversely proportional to the residence time of the measured dynamic process; thus the faster the motion, the broader the Lorentzian width. Background is assumed as a linear term *B(Q,E)* and *f* is a scaling factor including the well-known Debye–Waller factor for the H atoms*.* The intensities of the elastic *X(Q)* and quasielastic *(1-X(Q))* parts of the spectra are coupled. Hence, when a quasielastic process becomes observable at the measured time scale, the intensity of the elastic peak will decrease. Following the elastic incoherent intensity as a function of temperature (also called an elastic scan) is a typical approach to locate dynamic transitions. Therefore, an elastic scan was performed at BASIS to estimate the hydride ion dynamics in BaH_2_, as shown in Fig. 1. The elastic scan reveals three distinct areas: (i) low *T* region (from 300 K up to about 670 K), (ii) intermediate *T* region (from about 670 K up to about 775 K) (iii) high *T* region (*T* > 775 K)^9^. In the low *T* region, the elastic intensity decrease is about 50%, which can be correlated to the temperature dependency of the vibrational density of states (VDOS), discussed later in this work and in the SI. Around 670 K, the elastic intensity starts to decrease more rapidly, indicating the onset of observable hydride ion dynamics at the measured timescale. The sudden decrease around 775 K corresponds to the orthorhombic to hexagonal phase transition. Following this phase transition, the elastic intensity flattens out to a very small value, suggesting that the hydrogen in the structure becomes mobile in this high temperature phase. As we will discuss below, the hydride ions have very distinct dynamics on these three temperature regions.

**Figure 2:**
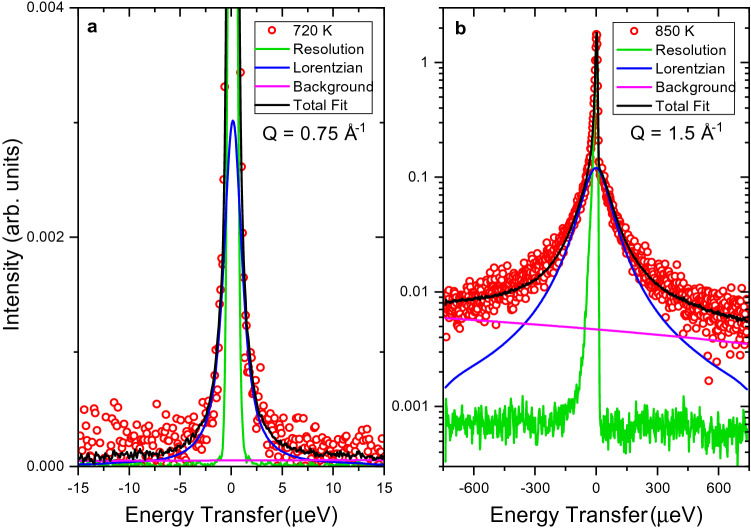
QENS spectra measured at (**a**) HFBS at *Q* = 0.75 Å^-1^ and *T* = 720 K (orthorhombic phase) and (**b**) measured at BASIS at *Q* = 1.5 Å^-1^ at T = 850 K (hexagonal phase) with the fit components shown.

A quasielastic signal is first observed at temperatures around 600 K but the signal is too weak to be accurately separated from the instrument resolution (see more details in the SI). The signal grows stronger with increasing temperature and can be readily resolved around 670 K. Upon the phase transition at 775 K, the quasielastic component increases drastically in both intensity and width. The QENS spectra was fitted with the Supplementary equation (S7) and two selected spectra are shown: Fig. 2a at *T* = 720 K for the low temperature orthorhombic phase and Fig. 2b at *T* = 850 K for the high temperature hexagonal phase.

**Figure 3:**
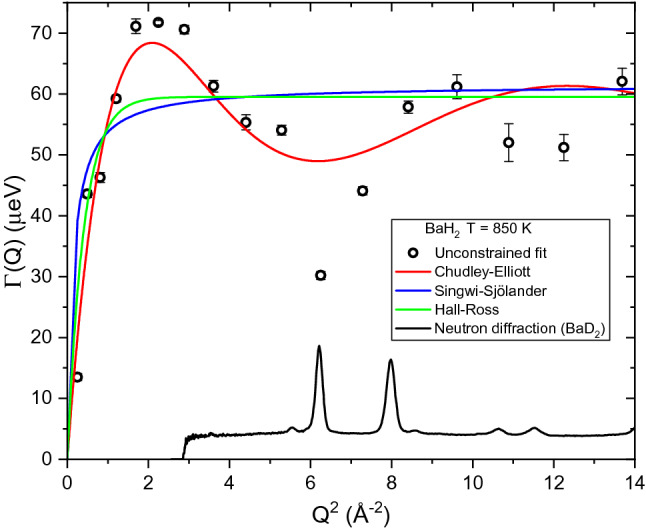
Comparison of QENS spectra fits with the constrained jump diffusion models (Supplementary equations (S7, S8 and S9)) to the unconstrained data fit (Eq. (1)) performed with *QClimax*. The three jump diffusion models are Chudley–Elliott, Singwi–Sjölander, and Hall–Ross^27–29^. Also shown is the neutron powder diffraction pattern for the hexagonal phase of BaD_2_ to highlight the Bragg peak positions.

The power of QENS arises from the ability to access both momentum and energy transfer domains at the same time, providing information about the characteristic diffusion processes. Jump diffusion models describe well-defined energy transfer relationships as a function of momentum transfer, *Q*, which yields details about the atomic scale diffusion process. As shown in Fig. 3, the Lorentzian HWHM extracted from fitting the QENS spectra with Eq. (1) can be plotted as a function of *Q*^2^ (black circles, unconstrained fit)_._ For comparison, we present three different jump diffusion models, namely the Chudley–Elliott^26,27^, Singwi–Sjölander^28^, and Hall–Ross^29^, that were fitted to the QENS spectra using global fitting algorithm built in *QClimax*, as described in detail in the SI. Clearly, the Singwi–Sjölander and Hall–Ross models are not in agreement with the unconstrained data fit compared to the Chudley–Elliott model. While the Chudley–Elliott model was initially derived for liquids with short range order, it has been used extensively to describe atoms diffusing in lattices, especially to describe ionic diffusion in solid electrolytes^4,30–32^. For these reasons, the Chudley-Elliott model was chosen to describe the QENS spectra of BaH_2_, and it takes the form,

2 $$\Delta E\left(Q\right)=\mathrm{\hbar }\Gamma \left(Q\right)=\frac{\mathrm{\hbar }}{\tau }\left[1-\frac{sin QL}{QL}\right]$$

where *L* is the jump length and *τ* is the residence time^26,27^. As described in detail in the SI, a global fitting procedure was then applied using *QCLIMAX* to constrain Eq. (1) to follow an ideal Chudley–Elliott behavior. The method of using *QCLIMAX* to apply a global fitting procedure with jump diffusion models has been demonstrated previously^33–35^. This procedure was performed by systematically fitting each QENS spectra at every *Q*-value simultaneously with the Lorentzian widths constrained to follow Eq. (2), resulting in Supplementary equation (S7). *Γ* has values on the order of few μeV in the orthorhombic phase while values are more than an order of magnitude larger in the hexagonal phase. Additional details about the data fits, including comparisons of fit results using *QCLIMAX* and *DAVE*^36^*,* are included in the SI. Since the data is well described by the Chudley–Elliott jump diffusion model, the motion can be attributed to the long-range translational diffusion of the hydride ions. Jump lengths and residence times were extracted and used to calculate diffusion coefficients (Supplementary equation (S4)). A summary of the fit parameters is displayed in Table 1.

**Table 1:** Jump lengths, residence times, and diffusion coefficients determined from QENS measurements.

Orthorhombic phase	T (K)	L (Å)	τ (ns)	D (10^–7^ cm^2^/s)
HFBS CCR	670	4.2 ± 0.2	1.7 ± 0.08	1.7 ± 0.2
	720	4.1 ± 0.1	1.0 ± 0.03	2.7 ± 0.2
	750	4.0 ± 0.1	0.7 ± 0.02	3.8 ± 0.2

Hexagonal phase	T (K)	L (Å)	τ (ps)	D (10^–5^ cm^2^/s)
BASIS Si (311) Furnace	770	3.1 ± 0.02	20.7 ± 0.1	0.8 ± 0.009
	790	3.1 ± 0.01	17.2 ± 0.07	0.9 ± 0.006
	810	3.1 ± 0.01	14.7 ± 0.06	1.1 ± 0.008
	830	3.1 ± 0.01	13.1 ± 0.04	1.2 ± 0.006
	850	3.1 ± 0.01	11.7 ± 0.05	1.4 ± 0.01
	870	3.1 ± 0.03	9.5 ± 0.07	1.7 ± 0.03
	920	3.1 ± 0.01	9.0 ± 0.04	1.8 ± 0.01

**Figure 4:**
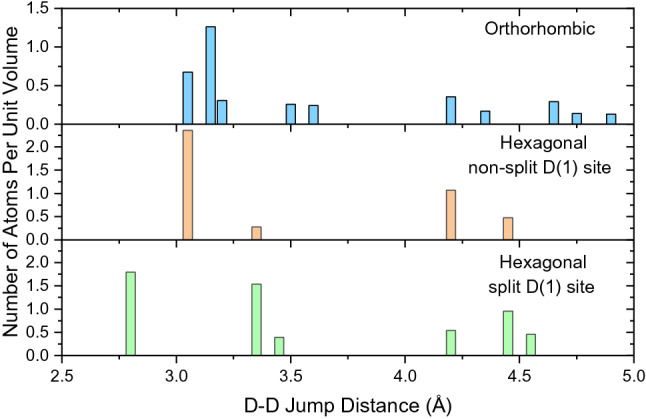
Deuterium-deuterium distances reported from neutron diffraction data for BaD_2_^1^. The orthorhombic and hexagonal phases were measured at *T* = 670 K and 883 K, respectively. The hexagonal phase distances are calculated for the D(1) atoms residing on the non-split 2d sites and split 4f. sites.

**Figure 5:**
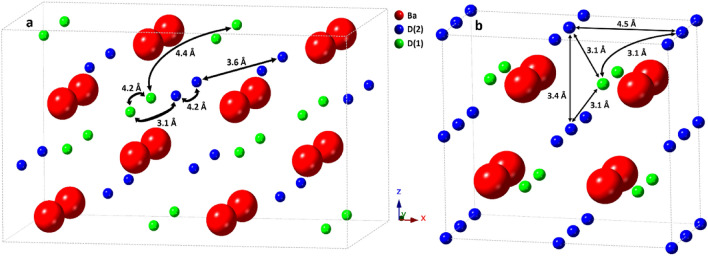
Crystal structures and potential diffusion pathways in BaD_2_ for the (**a**) low temperature orthorhombic phase at 670 K and the (**b**) high temperature hexagonal phase at 883 K. Additional jump lengths are also shown for comparison.

First, the diffusive motion in the orthorhombic phase will be discussed. This motion is observed as a narrow Lorentzian with *Γ* on the order of a few µeV. As seen in Table 1, the Chudley-Elliott model yields jump lengths in the orthorhombic phase with values around *L* = 4.0–4.2 Å. To compare this with the structural data, deuterium-deuterium (D-D) distances in BaD_2_ reported from neutron diffraction measurements are displayed in Fig. 4^1^. We do not expect the actual protium-protium (H–H) distances in BaH_2_ to be significantly different due to the difference in isotope; the unit cell volume for the deuterated systems is only smaller by about 0.438% at ambient conditions compared to the protonated systems. Due to the unique neutron scattering potential of hydrogen, protonated samples (BaH_2_) are used for dynamics investigations while deuterated samples (BaD_2_) are used for structural (neutron diffraction) measurements. For clarity, we will no longer dissociate between protium and deuterium, instead referring to both as hydrogen. Figure 4 shows that the shortest H–H distances in the orthorhombic structure can be separated into three main regions of approximately 3.1 Å, 3.6 Å, and 4.2 Å. The first region contains the shortest distances (3.1 Å), which corresponds to both H(1)-H(2) and H(1)-H(1) distances. The second region contains the next shortest distances (3.6 Å) due exclusively to H(2)-H(2) distances. The third region (4.2 Å) corresponds to both H(1)-H(1) and H(2)-H(2) distances. Therefore, the observed jump lengths in the orthorhombic phase correspond to jumps from H(1) to H(1) and/or from H(2) to H(2) sites along 4.2 Å jumps. Some potential jump pathways are shown in Fig. 5, along with other jump distances displayed for comparison. The diffusion pathway had been previously assumed to be along the (102) plane, where the H–H distances are the shortest, i.e. 3.1–3.2 Å^1^. The QENS data was fitted with one Lorentzian function (corresponding to one dynamic process) and a background term. It is possible that the background includes faster QENS contributions that are not distinguished. Thus, faster jumps, e.g. along the shortest available paths, may occur but are not observed on the timescale of the measurements done in this work. A study using a time-of-flight QENS spectrometer to access faster time-scales than backscattering spectrometers could help to find such jumps in BaH_2_. However, without evidence of these faster jumps, our current study suggests that H(1)-H(2) jumps are not occurring readily in the orthorhombic phase. Without the shorter jump processes, long-range translational diffusion would be greatly hampered in the orthorhombic phase. While computational modelling could shed some light on the energy landscape, the significant sub-stoichiometry of the hydrogen sublattice makes the modelling very difficult, if not impossible.

**Figure 6:**
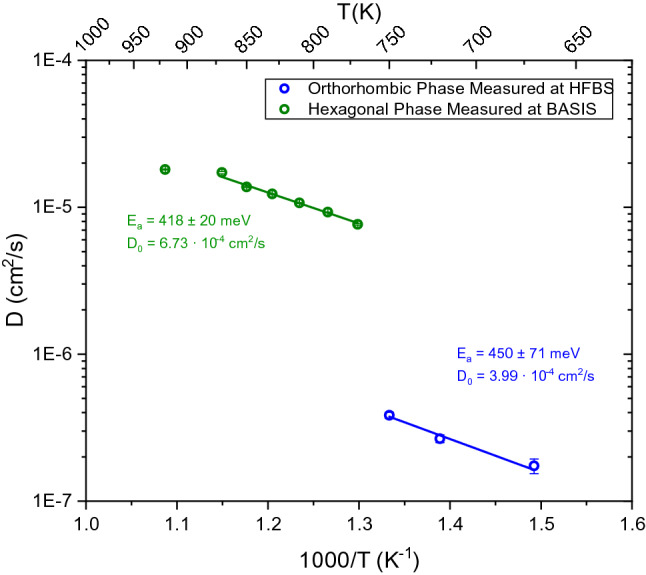
Arrhenius diagram for BaH_2_ corresponding to diffusion coefficients calculated from experimental QENS data at BASIS and HFBS. Solid lines are a linear fit of the data. Activation energies and preexponential diffusion coefficients are reported.

Next, we will discuss the diffusive motion in the hexagonal phase. As observed in Fig. 2, the quasielastic broadening increases drastically in the hexagonal phase, which required the use of the Si (311) crystal analyzers at BASIS enabling access to wider energy range compared to the Si (111) analyzers. While time-of-flight spectrometers would generally give access to even higher energy transfers to potentially resolve faster processes, the use of BASIS with the Si (311) analyzers is beneficial in this case as particularly high Q values can be measured up to 3.8 Å^−1^. Motions taking place on length scales of few Å, such as jump diffusion processes, can be resolved with higher accuracy when the Q-range can be extended. This can be crucial when plausible jump distances differ by only fractions of an Å from each other. The diffusion coefficients are displayed in the Arrhenius plot in Fig. 6 along with activation energies, *E*_*a*_, and the temperature independent preexponential diffusion coefficient, *D*_*0*_. The phase transition is evident around 775 K where the diffusion coefficients increase by over an order of magnitude. The diffusion process in the hexagonal phase has an activation energy of 418 meV, which is lower than the orthorhombic phase at 450 meV. A reduction in the activation energy for ionic transport is often observed following a transition to a higher symmetry phase. However, both the orthorhombic and hexagonal phases were reported to have the same activation energy from previous electrochemical impedance spectroscopy measurements (520 meV)^1^. Considering the large errors shown in Fig. 6, i.e. 450 ± 71 meV (orthorhombic) and 418 ± 20 meV (hexagonal), these values can be considered very similar in our study as well. The QENS value is notably lower than that from electrochemical impedance spectroscopy for the hexagonal phase. The activation energies calculated using these two techniques may be different because of the difference in time and length scales that each technique probes. For example, electrochemical impedance spectroscopy probes bulk diffusion over macroscopic length scales while QENS reveals information about diffusive motions on the angstrom scale. The jump lengths extracted from QENS are all very close to 3.1 Å, which is in perfect agreement with the shortest H–H distance (non-split site model), as seen in Fig. 4. This distance corresponds to H(1)-H(2) sites which are seemingly restricted in the orthorhombic structure. The H(1) sites now act as a ‘steppingstone’ for the diffusion process, allowing the hydrogen to diffuse in virtually any direction through the crystal structure along 3.1 Å jumps. Hence, the phase transition reduces the energy barrier that restricted the H(1)-H(2) jumps in the orthorhombic phase. As a result, the hydrogen is now allowed to diffuse using the shortest H–H distances. As expected, this produces residence times that are significantly shorter in the hexagonal phase, i.e. tens of picoseconds compared to nanoseconds. As mentioned previously, neutron powder diffraction measurements of the hexagonal phase suggested that the H(1) site is split, where it deviates from the high-symmetry 2d site and instead resides on the 4f site^1^. Recent total neutron scattering measurements also showed a slight improvement using this split site model^9^. This site splitting would further reduce the jump length from 3.1 Å to 2.8 Å. However, the QENS measurements consistently yield jump lengths of approximately 3.1 Å. This indicates that either QENS just observes the average jump length or that the hydrogen site isn’t split but just possesses a large thermal cloud. In addition, we have included a neutron powder diffraction pattern in Fig. 3 for BaD_2_ measured at T = 850 K to display the position of Bragg peaks in the QENS spectra. The Bragg peaks slightly decrease the value of the extracted Lorentzian widths, as seen in Fig. 3. Removing these *Q*-values from the fitting procedure did not significantly affect the extracted parameters. For example, when the *Q*-values that overlapped with Bragg peaks were removed from the fitting procedure, the corresponding jump length at 850 K was *L* = 3.03(9) Å, compared to *L* = 3.10(1) Å with the entire *Q*-range included. Hence, removing the Bragg peak contribution slightly reduces the jump length, but 2.8 Å jump lengths are still not observed.

**Figure 7:**
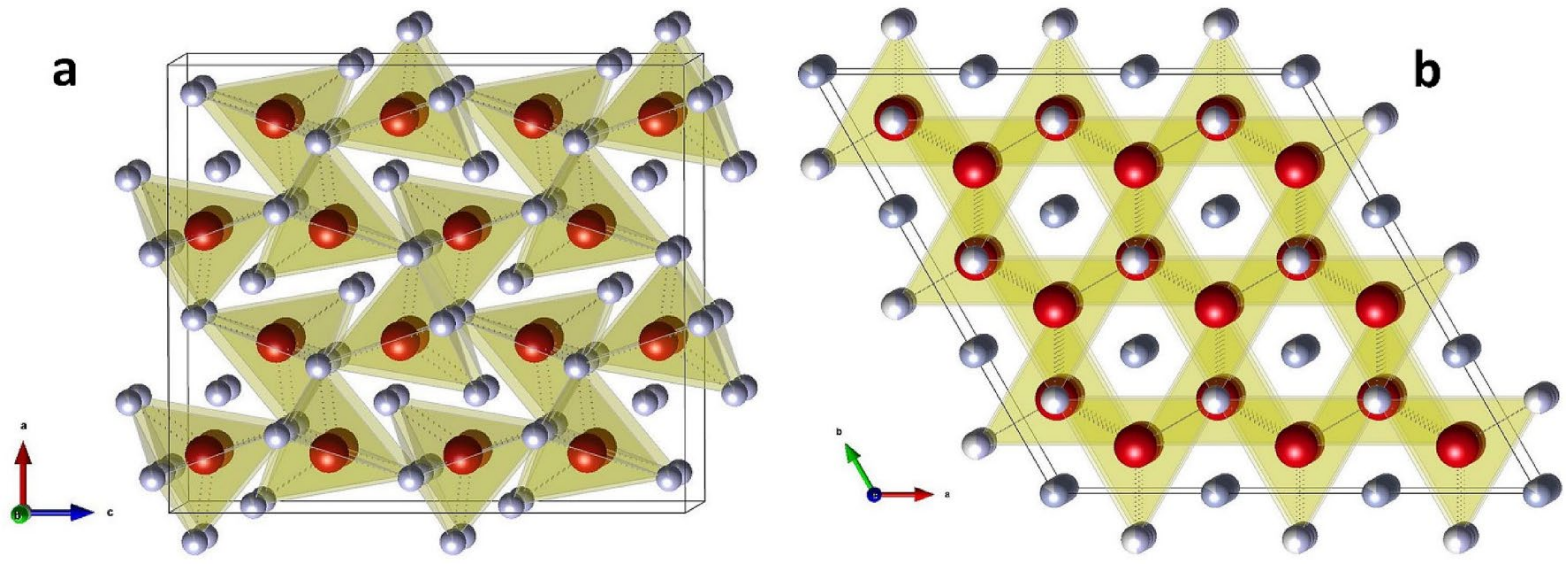
Crystal structure showing the tilt of the anionic polyhedra for the (**a**) orthorhombic and (**b**) hexagonal phases of BaD_2_.

The cotunnite to Ni_2_In-type phase transition has been known to occur in many types of materials, such as the fluorite-type compounds and Na_2_S. Comparisons between these two closely related structures have been described in detail previously^16,37,38^. The cations are positioned on a hexagonal close packed (hcp) lattice in both structures. However, the cotunnite structure is characterized by an orthorhombic unit cell because the cations deviate from the proper hcp positions. Six anions surround the cation forming a trigonal prism. In the cotunnite structure, three additional anions surround the cation (coordination number (CN) = 9) while in the Ni_2_In-type structure, five anions are present around the cations (CN = 11). As shown in Fig. 7, the disorder in the cation positions causes the adjacent anionic polyhedra to be tilted 25**°** with respect to each other. Following the phase transition, the cations are now properly aligned on the hcp sites which removes the tilt, resulting in a higher-symmetry hexagonal phase. While the long-range, global structure determined from Rietveld refinements would suggest the tilt no longer remains, total neutron scattering measurements have shown that it is likely that the anionic polyhedra in the hexagonal phase have some degree of reorientational freedom^9^. The polyhedra can fluctuate between various tilted configurations, which helps explain the existence of the split H(1) site. These dynamic structural fluctuations would be beneficial to the diffusion process by shortening the hydrogen jump length and reducing the energy barriers by increasing the free volume to diffuse through; hence another reason why enhanced diffusion occurs in this phase. The polyhedra would be largely locked in place in the orthorhombic phase but become orientationally mobile in the hexagonal phase. Anionic reorientational motion has been reported in complex hydrides, which serves to unlock fast ionic diffusion of cations in the material^39–41^.

These results have interesting ramifications for the lighter weight heavy alkaline earth hydrides of CaH_2_ and SrH_2_ because they possess the same cotunnite structure as BaH_2_ at ambient conditions. Neither CaH_2_ or SrH_2_ exhibit a temperature induced cotunnite to Ni_2_In-type phase transition (an unidentified phase transition occurs in SrH_2_ around 850** °C**)^42^, but a pressure induced transition of this type has been previously observed^43–45^. However, it could be possible to produce this type of temperature induced phase transition through chemical doping. The application of chemical doping to control phase transitions is used in the well-known compound yttria-stabilized zirconia^46^. This could potentially unlock fast hydrogen diffusion in a lighter weight hydride that would be more suitable for applications.

**Figure 8:**
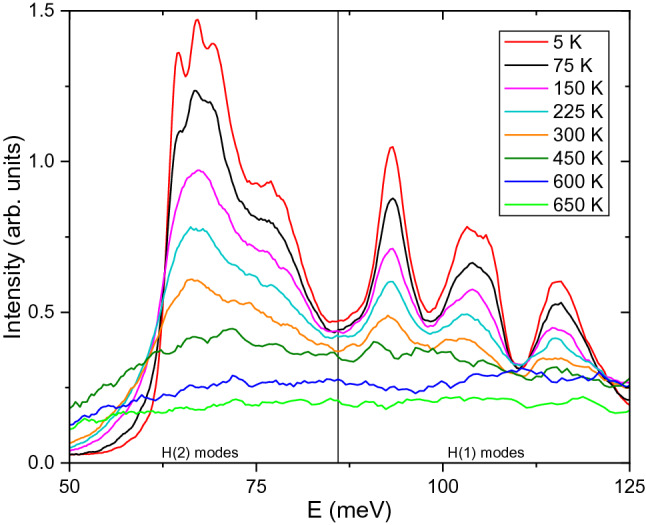
Inelastic neutron scattering spectra from 5 to 650 K for BaH_2_ measured at VISION. The optical phonon modes corresponding to H(1) and H(2) sites are labeled.

The vibrational density of states can be measured by powder INS to examine how bonding and local structure influences processes such as the hydrogen release mechanism. We conducted a temperature dependent INS investigation of BaH_2_ at VISION^23^, with the spectra shown in Fig. 8. More details of the temperature development of these modes are discussed in the SI and the Supplementary Figure S4. Since the hydrogen resides on two distinct crystallographic sites, their contributions to the INS spectra can be separated because they produce distinct vibrational modes. The neutron vibrational spectra of BaH_2_ have been studied previously^9,47–49^. The spectral region of interest for understanding the hydrogen vibrations is the Ba–H optical modes. The modes corresponding to the optical phonons of the H(2) atoms are located between 50 to 85 meV while modes for the H(1) atoms are located between 85 to 125 meV. In the orthorhombic structure, the H(2) atoms are positioned in the octahedral sites while the H(1) atoms are in the tetrahedral sites. However, due to the distorted structure, the hydrogen does not reside in the ideal positions in these cavities. For example, only 5 Ba atoms are coordinated to the H(2) sites in a square pyramidal geometry, rather than the ideal octahedral geometry. The H atoms are undergoing rapid thermal oscillations inside these tetrahedral and octahedral cavities. While they observe a largely static potential from the Ba sublattice, there is an additional rapidly fluctuating potential from the neighboring H sites due to the long-range ionic interaction. Therefore, the energy landscape is very complex in this system. To jump to the neighboring H sites, the H atoms need to overcome the potential barriers of the local environment. The sharp distinct modes at 5 K begin to decrease in intensity and broaden as temperature increases, as expected with Debye–Waller behavior. Softening of the vibrational modes can be noted at 450 K, and a small excess contribution is still present in the spectra for both the H(1) and H(2) optical modes. This indicates that the Ba-H modes remain intact but that the hydrogen is rapidly bouncing around the tetrahedral and octahedral cavities. The modes continue to lose more intensity and become more dispersed around 600 K indicating that the hydrogen now likely possesses enough energy to begin undergoing long-range translational diffusion. This is the same temperature that a hint of a quasielastic signal is first observed using QENS. While the modes associated with the H(2) sites have completely disappeared at 600 K, there is still a very small amount of excess intensity remaining in the two highest energy modes of the H(1) region (approximately 105 meV and 115 meV). As expected with H(2) being the lower energy site, the H(2) atoms likely begin diffusing at a lower temperature compared to the higher energy H(1) sites. The temperature dependence of the integrated intensities for both the H(1) and H(2) optical modes are shown in Supplementary Figure S4. This shows that the intensity of the lower energy H(2) sites decreases faster than the higher energy H(1) sites. While the behavior is similar for both sites, the temperature dependence of the H(2) modes are shifted to lower temperatures by approximately 70 K. This further supports our QENS results that the H(2) likely begins diffusing around 600 K, while the QENS measurements at *T* ≥ 670 K involve the diffusion of both hydrogen sites.

## Conclusions

In this work, we studied the unique conduction mechanism of pure hydride ion diffusion in barium hydride using QENS. Our results show that the hydride ions undergo jump diffusion between various hydrogen lattice sites, as explained by the Chudley-Elliott jump diffusion model. We suggest the preferred jumps in the lattice for both the low and high temperature structures, with each phase showing very different dynamics. In the low temperature orthorhombic phase, we observed jump lengths of 4.2 Å, which corresponds to distances between both H(2)-H(2) and H(1)-H(1) sites. Despite the shorter distances of 3.1 Å and 3.6 Å in the structure, we do not observe such jumps. In the high temperature hexagonal phase, jump lengths were found to be around 3.1 Å, which corresponds to jumps between H(1)-H(2) sites that were previously restricted in the orthorhombic phase. This change allows the hydrogen to diffuse efficiently through the shortest jump distances. Furthermore, the jump rate increases by an order of magnitude upon the phase transition promoting the faster diffusion. Hydrogen site splitting and dynamic fluctuations of anionic polyhedra in the hexagonal phase serves as a key for unlocking fast hydrogen diffusion. These results have interesting implications for the lighter weight hydrides that crystallize in the cotunnite structure. A similar phase transition, as occurs in BaH_2_ at high temperature, could potentially be obtained in other metal hydrides by applying pressure or by doping, which could in turn lead to fast hydrogen diffusion. This investigation served to demystify the role that a structural phase transition plays in transforming a solid-state material with modest kinetics into a fast-ionic conductor of hydrogen.

## Supplementary Information

Supplementary Information.

## Data Availability

The datasets generated and/or analysed during the current study are available from the corresponding author on reasonable request.
