# Supplementary material for: Uncovering the hydride ion diffusion pathway in barium hydride via neutron spectroscopy
Source: Sci Rep. 2022 Apr 13;12:6194. doi: 10.1038/s41598-022-10199-8 (PMC9007959; doi:10.1038/s41598-022-10199-8)
Supplement: Supplementary file 1 — Supplementary Information. [file 41598_2022_10199_MOESM1_ESM.pdf]

Supplementary Information: Uncovering the Hydride Ion Diffusion Pathway in Barium Hydride via Neutron Spectroscopy

Eric Novak,[†,‡,§], Luke Daemen[‡], Anibal Javier Ramirez-Cuesta[‡], Yongqiang Cheng[‡], Robert Smith[||], Takeshi Egami[†,¶,#], Niina Jalarvo*,[‡,§]*

[†]*Department of Materials Science and Engineering, University of Tennessee, Knoxville, TN 37996, United States*

[‡]*Neutron Sciences Directorate, Oak Ridge National Laboratory, Oak Ridge, TN 37831, United States*

[§]*Jülich Centre for Neutron Science, Forschungszentrum Jülich GmbH, 52425 Jülich, Germany*

[||]*Computing & Computational Sciences Directorate, Oak Ridge National Laboratory, Oak Ridge, TN 37831, United States*

[¶]*Materials Science and Technology Division, Oak Ridge National Laboratory, Oak Ridge, TN 37831, United States*

[#]*Department of Physics and Astronomy, University of Tennessee, Knoxville, TN 37996, United States*

**Corresponding author: *Eric Novak:* novakec@ornl.gov, **Niina Jalarvo:* jalarvonh@ornl.gov

Experimental Details

Quasielastic Neutron Scattering: QENS measurements were performed using the backscattering spectrometers BASIS[1] at Oak Ridge National Laboratory and HFBS at the National Institute of Standards and Technology (NIST).[2] Data was collected in two different experiments: (1) closed cycle refrigerator (CCR) at HFBS and (2) using a vacuum furnace at BASIS. All sample handling was performed in a helium glove box to avoid exposure to atmospheric water and oxygen. Cylindrical titanium sample cans were used at HFBS with gold O-ring seals. At HFBS, 1.5 g of BaH_2 was loaded into an aluminum foil packet before being loaded into the titanium sample can. At BASIS, the sample was measured in a 5 mm diameter quartz NMR tube with m = 0.97 g of sample and measured under a helium gas atmosphere maintained at atmospheric pressure. Two different measurement configurations were used at BASIS using the Si(111) and Si(311) analyzers. The Si(111) analyzers provide an accessible Q-range from 0.2 to 2.0 $Å^{-1}$, an energy range of ± 100 μeV, and an energy resolution of FWHM = 3.5 μeV. The Si(311) analyzers provide an accessible Q-range from 0.4 to 3.8 $Å^{-1}$, an energy range of ± 740 μeV, and an energy resolution of FWHM = 15 μeV. At BASIS, the instrumental resolution function was measured at T = 300 K. Shorter measurements for the elastic intensity scan were taken every 10 K between 300K and 880 K while longer scans with higher statistics suitable for detailed QENS data analysis were measured between 340 K and 920 K. At HFBS, measurements were conducted using the standard instrument configuration with an accessible Q-range from 0.25 to 1.75 $Å^{-1}$, an energy range of ± 16 μeV, and an energy resolution of FWHM = 0.8 μeV. Empty vanadium cans were used to measure the instrumental resolution function. Empty can subtractions were performed for the HFBS measurements but not for BASIS. We have performed empty can measurement at BASIS with a similar sample environment set up at a number of temperatures and could not observe any visible QE contribution. However, empty can subtraction could help to distinguish any wide, flat, or low intensity components that are seemingly part of the background. Thus, it is possible that we would have missed such a QE component.

Inelastic Neutron Scattering: Vibrational spectroscopy measurements were performed at VISION at ORNL.[3] VISION is highly sensitive to hydrogen displacements and has an accessible energy range from the elastic limit up to 500 meV with an energy resolution of $\Delta E/E \approx$ 1-2% across the entire energy range. For the low temperature measurements between 5 K and 300 K, a 0.53 g BaH_2 sample was measured in an 8 mm diameter vanadium PAC can in a CCR. Higher temperature measurements from 300 K to 650 K were measured using a cylindrical stainless-steel sample can with a copper O-ring seal. The sample (m = 1.4 g) was suspended in an annular copper foil packet. Data reduction was performed using Mantid.[4] Empty sample holders were measured at various temperatures and subtracted from the data.

Neutron Powder Diffraction: NPD measurements were performed at NOMAD at ORNL.[5] BaD_2 was measured at 850 K in a vacuum furnace. A 0.925 g sample was loaded into a 5 mm diameter quartz NMR tube. An empty sample holder was used for background subtraction.

Data Analysis Methods

The QENS data was analyzed based on the following formalism:

The measured signal, S(Q,E), is fit using:

$$S_{inc}(Q,E) = f\left[X(Q)\delta(E) + \left(1 - X(Q)\right)S_{qe}(Q,E)\right] \otimes R(Q,E) + B(Q,E) \qquad (S1)$$

Here, X(Q) is the fraction of elastic scattering, known as the elastic incoherent structure factor (EISF), δ(E) is the elastic signal, and $S_{qe}(Q,E)$ is the model representing the quasielastic scattering. These terms are numerically convoluted with the resolution

function R(Q,E). B(Q,E) is the linear background term. Only one quasielastic process is observed at HFBS and BASIS: at HFBS, the motion corresponds to the slower diffusion process in the orthorhombic phase while the quasielastic process observed at BASIS corresponds to the faster diffusion process in the hexagonal phase. Therefore, this data is fit with a single Lorentzian model,

$$S_{qe}(Q,E) = \frac{1}{\pi}\frac{\Gamma(Q)}{E^2 + \Gamma^2(Q)} \qquad (S2)$$

where Γ is the Lorentzian half-width at half-maxima (HWHM).

The Lorentzian broadening at low momentum transfers follows the characteristic DQ^2 dependence associated with long-range translational diffusion. At higher Q, the broadening deviates from the DQ^2 dependence, which yields information about the nature of the diffusion process. Different jump-diffusion models were tested, as shown in Fig. 3 in the main text. The Chudley-Elliott jump diffusion model[6,7] best describes the QENS data,

$$\Delta E(Q) = \Gamma(Q) = \frac{\hbar}{\tau}\left[1 - \frac{\sin QL}{QL}\right] \qquad (S3)$$

where L is the jump length and τ is the residence time. In this model, the particle resides in the same average position, only undergoing thermal oscillations about this lattice site. After remaining in that position for a characteristic residence time (τ), the particle jumps over a discrete distance to another available site. The diffusion coefficient for the Chudley-Elliott model can be calculated using the following equation.[6,7]

$$D = \frac{L^2}{6\tau} \qquad (S4)$$

After plotting the obtained diffusion coefficients in an Arrhenius format, the Arrhenius equation

$$\ln(D) = -\frac{E_a}{R}\left(\frac{1}{T}\right) + \ln(D_0) \qquad (S5)$$

was applied to obtain activation energies, E_a, and the temperature independent preexponential diffusion coefficient, D_0.

The QENS data fits were performed using the QCLIMAX package within ICE-MAN, the Integrated Computational Environment-Modeling & Analysis for Neutrons. ICE-MAN has been recently developed at ORNL for analysis of neutron scattering data. The unconstrained initial fits showed a clear Chudley-Elliott diffusive behavior. Therefore, the Lorentzian widths were further constrained to follow the Chudley-Elliott model of equation (S3) (comparison of fits with DAVE and QCLIMAX are shown later). The software fits the QENS spectra at every Q value simultaneously, effectively fitting $S_{qe}(Q,E)$ to the following quasielastic model

$$S_{qe}(Q,E) = \frac{1}{\pi}\frac{\frac{\hbar}{\tau}\left[1 - \frac{\sin QL}{QL}\right]}{E^2 + \left(\frac{\hbar}{\tau}\left[1 - \frac{\sin QL}{QL}\right]\right)^2} \qquad (S6)$$

and determining by minimization the parameters L and τ.

The QENS spectra is therefore fit using the following equations for a global fitting procedure that is constrained to follow the Chudley-Elliott (equation (S7)), Singwi-Sjölander (equation (S8)), and Hall-Ross (equation (S9)) models.[6-9]

$$S(Q,E) = f\left[X(Q)\delta(E) + \left(1 - X(Q)\right)\frac{1}{\pi}\frac{\frac{\hbar}{\tau}\left[1 - \frac{\sin QL}{QL}\right]}{E^2 + \left(\frac{\hbar}{\tau}\left[1 - \frac{\sin QL}{QL}\right]\right)^2}\right] \otimes R(Q,E) + B(Q,E) \qquad (S7)$$

$$S(Q,E) = f\left[X(Q)\delta(E) + \left(1 - X(Q)\right)\frac{1}{\pi}\frac{\frac{\hbar DQ^2}{1 + DQ^2\tau}}{E^2 + \left(\frac{\hbar DQ^2}{1 + DQ^2\tau}\right)^2}\right] \otimes R(Q,E) + B(Q,E) \qquad (S8)$$

$$S(Q,E) = f\left[X(Q)\delta(E) + \left(1 - X(Q)\right)\frac{1}{\pi}\frac{\frac{\hbar}{\tau}\left[1 - e^{-DQ^2\tau}\right]}{E^2 + \left(\frac{\hbar}{\tau}\left[1 - e^{-DQ^2\tau}\right]\right)^2}\right] \otimes R(Q,E) + B(Q,E) \qquad (S9)$$

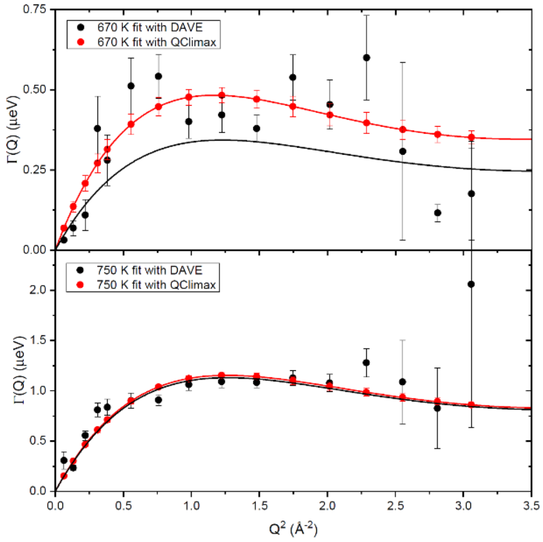

Figure S1. Q-dependence of the HWHM Γ(Q) for HFBS at 670 K and 750 K. The black circles are the result of fitting the QENS spectra with *DAVE* while the red circles are the constrained *QClimax* fits. The solid lines are fits of the Chudley-Elliott model.

Comparison of *DAVE* vs. *QCLIMAX*

A comparison of the Q-dependence of the Lorentzian widths fit with *QCLIMAX* vs. *DAVE*[10] is shown in Fig. S1. DAVE was used to fit each Q value individually with equation (S1) and the Lorentzian width was extracted from these fits (black circles). The resulting widths were then fit manually with the Chudley-Elliott model, equation (S3) (black line). Next, the global fitting procedure (Eq. S7) that uses *QCLIMAX* to fit the entire range of Q-values simultaneously to the Chudley-Elliott model was applied (red line). The fit of the Chudley-Elliott model is almost identical with respect to *QCLIMAX* and *DAVE* for the 750 K data. The errors calculated with *QCLIMAX* are noticeably smaller which yields more accurate jump lengths and residence times. The benefits of *QCLIMAX* becomes apparent for the 670 K data, which is far more difficult to analyze due to the smaller quasielastic intensity and narrower broadening. By constraining the fitting algorithm to follow a Chudley-Elliott behavior, *QCLIMAX* can produce an excellent fit of the data that also agrees well with the widths obtained in the DAVE fits.

A comparison of the fit results from *QCLIMAX* for an unconstrained fit vs. a fit constrained to follow the Chudley-Elliott jump diffusion model is shown in Fig. S2 for T = 850 K. The unconstrained fit yields widths that follow a clear Chudley-Elliott

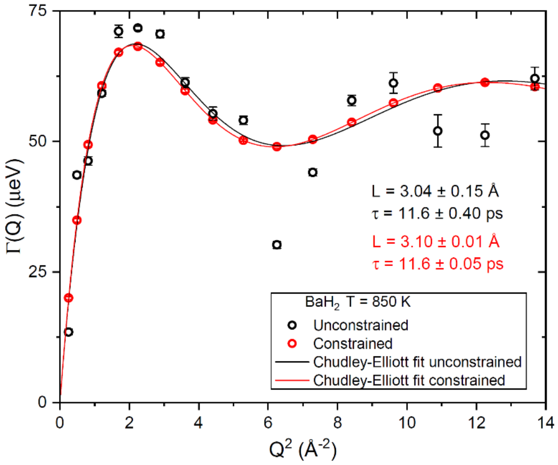

Figure S2. Comparison of the Lorentzian widths at T = 850 K calculated from an unconstrained fit vs. a fit constrained to follow the Chudley-Elliott jump diffusion model. Jump lengths and residence times are given.

relationship. The jump lengths and residence times extracted from the fits are also displayed in Fig. S2. Clearly, the errors are reduced using the constrained fit. Both the unconstrained and constrained fits yielded similar results, verifying that the assumptions in our global fitting procedure are justified and serve to increase the accuracy of our results.

HFBS Spectra Comparison

In the main text, it was mentioned that a quasielastic broadening was first observed around 600 K, but that the width was too narrow compared to the instrumental resolution to be reliably extracted. This can be observed in Figure S3, where the 600 K spectrum exhibits a slight broadening and continuously broadens with increasing temperature. The Lorentzian widths can first be reliably extracted beginning with the 670 K measurement. All the spectra in Figure S3 are normalized to unity (with respect to the elastic peak) to demonstrate the amount of quasielastic broadening with temperature.

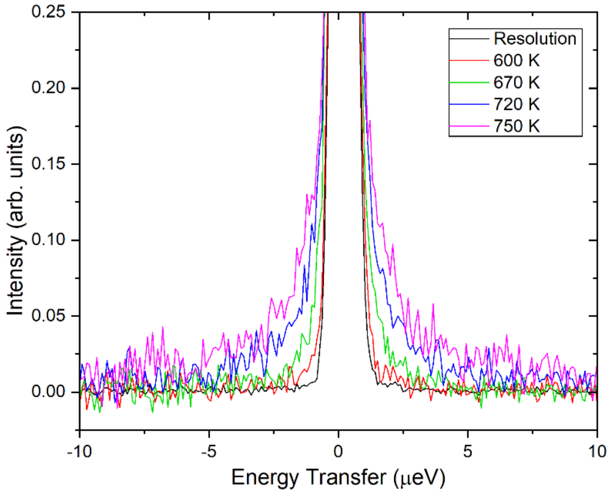

Figure S3. QENS spectra measured at HFBS over a temperature range of 600 – 750 K. The resolution function was measured at ambient conditions using a vanadium can.

INS Temperature Dependence

The temperature dependence of the H(1) and H(2) optical modes are shown in Figure S4. This was calculated by integrating the peak intensities over the entire H(1) and H(2) mode regions (H(2): 55–85 meV, H(1): 86–125 meV) across the temperature range of 5–650 K. Lastly, a 2nd order polynomial was applied to obtain a general fit of the data to quantify the temperature dependence of the optical modes, as shown as solid lines of Figure S4. As can be observed, the lower energy H(2) modes lose intensity faster at lower temperatures, but at higher temperatures both the H(1) and H(2) modes experience similar behavior. The H(2) modes follow this trend at a lower temperature of approximately 70 K compared to the higher energy H(1) sites. This can be interpreted that the H(2) sites would likely gain enough energy to overcome the thermal barriers for diffusion at a lower temperature compared to the H(1) sites.

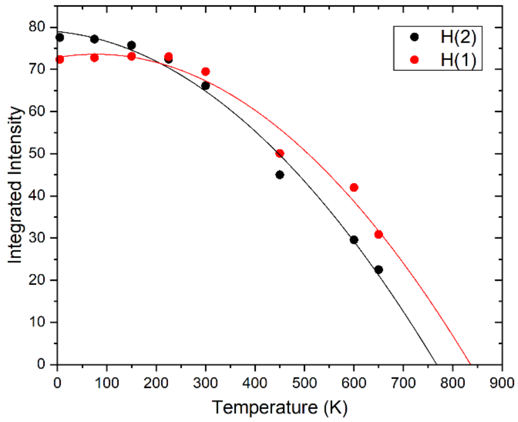

Figure S4. Temperature dependence of the integrated intensities for the H(1) and H(2) optical modes measured with INS. The solid lines represent a 2nd order polynomial fit of the data.

References

1 Mamontov, E. & Herwig, K. W. A time-of-flight backscattering spectrometer at the Spallation Neutron Source, BASIS. *Rev. Sci. Instrum.* **82**, 085109, doi:10.1063/1.3626214 (2011).

2 Meyer, A., Dimeo, R. M., Gehring, P. M. & Neumann, D. A. The high-flux backscattering spectrometer at the NIST Center for Neutron Research. *Rev. Sci. Instrum.* **74**, 2759-2777, doi:10.1063/1.1568557 (2003).

3 Seeger, P. A., Daemen, L. L. & Larese, J. Z. Resolution of VISION, a crystal-analyzer spectrometer. *Nucl. Instrum. Methods Phys. Res. A* **604**, 719-728, doi:https://doi.org/10.1016/j.nima.2009.03.204 (2009).

4 Arnold, O. *et al.* Mantid—Data analysis and visualization package for neutron scattering and μ SR experiments. *Nucl. Instrum. Methods Phys. Res. A* **764**, 156-166, doi:https://doi.org/10.1016/j.nima.2014.07.029 (2014).

5 Neuefeind, J., Feygenson, M., Carruth, J., Hoffmann, R. & Chipley, K. K. The Nanoscale Ordered MAterials Diffractometer NOMAD at the Spallation Neutron Source SNS. *Nucl. Instrum. Methods Phys. Res. B* **287**, 68-75, doi:https://doi.org/10.1016/j.nimb.2012.05.037 (2012).

6 Chudley, C. T. & Elliott, R. J. Neutron Scattering from a Liquid on a Jump Diffusion Model. *Proc. Phys. Soc.* **77**, 353 (1961).

7 Bée, M. *Quasielastic Neutron Scattering*. (Hilger, 1988).

8 Hall, P. L. & Ross, D. K. Incoherent neutron scattering functions for random jump diffusion in bounded and infinite media. *Mol. Phys.* **42**, 673-682, doi:10.1080/00268978100100521 (1981).

9 Singwi, K. S. & Sjölander, A. Diffusive Motions in Water and Cold Neutron Scattering. *Phys. Rev.* **119**, 863-871 (1960).

10 R.T. Azuah, L. R. K., Y. Qiu, P.L.W. Tregenna-Piggott, C.M. Brown, J.R.D. Copley, and R.M. Dimeo. DAVE: A comprehensive software suite for the reduction, visualization, and analysis of low energy neutron spectroscopic data. *J. Res. Natl. Inst. Stan. Technol.* **114** (2009).
